# Assessment of Knowledge and Practices regarding Injection Safety and Related Biomedical Waste Management amongst Interns in a Tertiary Care Teaching Hospital, Delhi

**DOI:** 10.1155/2014/670861

**Published:** 2014-10-28

**Authors:** Anita Shankar Acharya, Priyanka, Jyoti Khandekar, Damodar Bachani

**Affiliations:** Department of Community Medicine, Lady Hardinge Medical College, New Delhi 110001, India

## Abstract

Injuries caused by needle sticks and sharps due to unsafe injection practices are the most common occupational hazard amongst health care personnel. The objectives of our study were to determine the existing knowledge and practices of interns and change in their level following an information education and communication (IEC) package regarding safe injection practices and related biomedical waste management and to determine the status of hepatitis B vaccination. We conducted a follow-up study among all (106) interns in a tertiary care teaching hospital, Delhi. A predesigned semistructured questionnaire was used. IEC package in the form of hands-on workshop and power point presentation was used. A highly significant (*P* < 0.001) improvement in the knowledge of interns was observed after intervention with respect to the “three criteria of a safe injection” and cleaning of injection site. Thus, the baseline knowledge of interns was good in certain aspects of injection safety, namely, diseases transmitted by unsafe injections and their prevention. We conclude that IEC intervention package was effective in significantly improving the interns' knowledge regarding safe injection practices and biomedical waste management. Almost two-thirds of interns were immunised against hepatitis B before the intervention and this proportion rose significantly after the intervention.

## 1. Introduction

Injection use is one of the most important medical procedures used globally, mostly for administration of drugs and immunization. In a developing country like India, injection safety is a major concern. Unsafe injection practices put patients and healthcare providers at risk of infectious and noninfectious adverse events and have been associated with a wide variety of procedures and settings. This harm is preventable if we follow safe injection practices [1].

“An injection is said to be safe if it does not harm the recipient, does not expose the provider to avoidable risk, and does not result in wastes that is dangerous for the community.” (WHO, 2004) [2].

Injuries caused by needle sticks and sharps due to unsafe injection practices are the most common occupational hazard amongst health care personnel worldwide. The health care workers include doctors, nurses, laboratory technicians, dentists, phlebotomists, and refuse workers. The National Institute of Occupational Safety and Health (NIOSH) defines needle stick injury (NSI) as “Injuries that are caused by needles such as hypodermic needles, blood collection needles, intravenous (IV) stylets and needles used to connect part of IV delivery systems” [3].

The World Health Organization estimates that about 3 million needle stick injuries occur every year with 90% occurring in developing countries [4]. These needle stick injuries pose a serious health problem particularly for transmission of blood borne viruses like HIV, hepatitis B, and hepatitis C. The risk of HIV transmission without prophylaxis is 0.3% from percutaneous injury [3]. For unvaccinated healthcare workers, the risk from single needle sticks or cut exposure to HBV-infected blood ranges from 6 to 30%, depending on the hepatitis B antigen status of the source individual [5]. WHO estimates that annually 21 million hepatitis B infections, 2 million hepatitis C infections, and 260,000 HIV/AIDS cases may be caused by reuse of syringes and needles without sterilization accounting for 40%, 32%, and 5%, respectively [6].

An effective vaccine is available against hepatitis B infection but none is available against HIV or hepatitis C. Hence it becomes all the more pertinent to reenforce safe injection practices amongst the medical personnel.

Medical interns who begin their clinical career after completing their graduation are exposed to blood and blood products in various settings like immunization sessions, drawing blood samples, assistance during surgery, conducting deliveries, and so forth. It is important that they are fully aware of safe injection practices and related biomedical waste management. Even before starting their internship, medical students face similar type of exposures during their clinical postings in the departments of obstetrics and gynecology, medicine, surgery, accident and emergency, orthopedics, pediatrics, and so forth, thus making them vulnerable to deadly infections.

Thus, safe injection practice is critically important and its lack poses a major occupational health hazard for health care professionals. We chose to conduct this study on medical interns because they have less clinical experience and are more at risk of unsafe injection practices. There is a dearth of intervention based published studies on knowledge of interns on injection safety in India. With this background, we planned this study with the following objectives. 

*Objectives*

1. The aim is to determine the existing knowledge of medical interns regarding safe injection practices and related biomedical waste management.
2. The aim is to measure the change in the level of awareness following an IEC package regarding safe injection practices and related biomedical waste management amongst interns.
3. The aim is to determine the status of hepatitis B vaccination among the interns.

## 2. Material and Methods

*Study Design*. We conducted a follow-up study. 

*Study Period*. It was one year from December 31, 2011 to December 31, 2012. 

*Study Area*. The study area was a tertiary care teaching hospital and medical college, New Delhi, India, which is situated in central part of Delhi. This is one of the premier medical schools which started in 1916. This medical college had an intake of 130 students for the course of MBBS of four and half years' duration. 

*Study Population and Sample Size*. In India, the Bachelor of Medicine and Bachelor of Surgery (M.B.B.S) professional course consists of a four and half years' degree course consisting of four professional examinations followed by one year of compulsory rotatory internship in all major disciplines in which their bedside clinical skills are enhanced. After successful completion of internship, the M.B.B.S degree is awarded to them. So, the study population comprised of all the medical interns (106) who were to begin their compulsory rotatory internship for a period of one year from January 1, 2012 to December 31, 2012. A total of 106 interns were enrolled for the study. Out of them 101 completed the posttest assessment questionnaire. 

*Study Instruments*. A predesigned semistructured questionnaire was developed using previous published reports on injection safety [6–10] and pretested in the outgoing batch of interns in November 2011. IEC package in the form of hands, on workshop, power point presentation, video-films, charts on injection safety, and biomedical waste management was used. The areas covered in all the above IEC package included the “three criteria of safe injection” by WHO which are as follows: no harm to the recipient, no harm to the provider, no harm to the community, diseases transmitted by unsafe injection, vaccines to prevent blood borne infections, knowledge about autodisable (AD syringes), exposure to needle stick injuries, steps for prevention of needle stick injuries, postexposure prophylaxis against HIV, hepatitis B vaccination, disposal of injection related biomedical waste, and so forth. 

*Methodology*. As a part of skill development of interns, theory classes and practical supportive supervision with respect to safe injection practices and biomedical waste management are done on a regular basis in their two months posting of community medicine every year. We tried to analyze this regular activity. A common briefing is planned for all the interns every year before the start of their internship training which was held for this batch on December 31, 2011. The purpose of the study was explained and informed consent of the entire group was obtained. They were administered a predesigned, standardized, self-administered pretested questionnaire for the baseline assessment regarding their awareness of safe injection practices. The questionnaire consisted of questions related to all the topics covered in the IEC package as mentioned in the paragraph above under “Study Instruments”. A batch of 18–20 interns was posted in the Department of Community Medicine for a period of two months starting from January 1, 2012. In this way, six batches (A to F) rotated in the department in a period of one year from January 1, 2012.

We also conducted a one-day workshop in first week of January 2012 for the entire batch of interns after the pretest to sensitize them regarding safe injection practices and biomedical waste management. Subsequently, in the first week of their posting in community medicine, each batch (A to F) was given an intervention package consisting of a power point presentation and a video-film on safe injection practices and biomedical waste management. Along with it charts and posters were shown by a single investigator during their period of posting in the department throughout the year. They also got hands-on experience in safe injection practices and biomedical waste management during their postings in health centers and during immunization sessions. In this way, they were exposed to repeated IEC to reinforce their knowledge and skills required for injection safety and biomedical waste management.

In the last week of their two months posting in the Department of Community Medicine, they were again given the same self-administered questionnaire as a posttest and their change in the level of knowledge was assessed.

*Statistical Study.* Data were entered and analyzed in statistical software package SPSS version 16. Descriptive statistics were used to describe the distribution of all variables. Bivariate analysis using McNemar's chi-square was used to examine the association between each dependent variable at pretest and posttest.

## 3. Results

Out of the total 106 pretest and posttest questionnaires, 101 were complete and were analyzed with a response rate of 95.28%.

Almost all the interns (98%) had good knowledge in the preintervention assessment, about diseases which can be contracted by unsafe injections, namely HIV, hepatitis B and hepatitis C. Majority (92.1%) knew about hepatitis B vaccine to prevent infection with this virus. All the interns (100%) knew about the three most commonly used routes for giving injection. Most of the interns (93.1%) had good knowledge about life-saving drugs present in an emergency tray which is extremely important for management of emergencies (like anaphylaxis) following administration of injections [11]. Almost two-thirds knew correctly about the postexposure prophylaxis (PEP) against HIV (74.3%) and the time period after which PEP is not effective (76.2%). Only 59.4% interns had knowledge about not using spirit swab before administration of any live vaccine (BCG, measles) (Table 1).

However, the knowledge was poor for some aspects of injection safety. Only 23% interns knew correctly about all the three criteria for a safe injection and only 10% knew correctly the correct method of opening a glass ampoule and correct handling of syringe after giving injection. Only 24% interns were aware of the do not's after a needle stick injury. Knowledge was also poor regarding biomedical waste management related to injections. Only 17% knew the correct method of disposal of an empty vial. Nearly one in four (26.7%) of them knew correct method to dispose gloves.

Tables 1 and 2 depict the comparison of the pre- and postintervention assessment of knowledge and skills' regarding injection safety and related biomedical waste management, respectively. A highly significant (*P* < 0.001) improvement in the knowledge of interns was observed after intervention with respect to the “three criteria of a safe injection,” use of autodisabled syringe, cleaning of injection site, not to use spirit swab before administering any live vaccine, procedure to open a glass ampoule, precautions and do's and do not's after NSIs, reporting of NSI, ideal time for initiating postexposure prophylaxis (PEP), handling needle and syringe after giving injection, and disposal of injection related waste.

Other aspects in which significant improvement in knowledge of interns was observed after IEC intervention were knowledge about common complications after giving injection (*P* = 0.03), time after which PEP is not effective (*P* = 0.01) and disposal of needle cap (*P* = 0.04). However, there was no significant improvement in knowledge regarding “postexposure prophylaxis after NSI” (*P* = 0.21) and disposal of broken ampoule (*P* = 0.28).

### 3.1. Hepatitis B Immunization Status (Table 3)

Baseline (pretest) information regarding the immunization status of interns against hepatitis B was collected. It was observed that 67 (66.3%) interns had completed the three-dose HBV vaccination schedule, 22 (21.8%) were partially immunized with either one or two doses, and remaining 12 (11.9%) were not immunized against hepatitis B. After the intervention, there was significant (*P* < 0.001) improvement in their immunization status. As many as 78 (78.2%) interns had completed the 3-dose HBV vaccination schedule, 20 (19.8) were partially immunized, and only three interns remained unimmunized.

### 3.2. Exposure to Needle Sticks Injury

At the baseline stage, information was collected on history of needle stick injuries in the past, that is, during the four and half years of M.B.B.S. course. Six interns (5.9%) reported needle stick injury in the past before the start of their internship; four of them had one needle stick injury while the other two had inflicted it twice. Information was also collected about needle stick injuries which occurred after the start of internship. Twenty-eight (27.7%) interns reported NSI with 22 interns having one exposure, 5 interns had two exposures, and one intern had injury thrice during the period of their internship.

## 4. Discussion

As a medical student in the M.B.B.S curriculum, students are taught about safe injection practices and biomedical waste management. It was seen that nearly all (98%) medical interns had good knowledge about the diseases transmitted by unsafe injections, namely, HIV, hepatitis B, and hepatitis C. All the interns knew about the three most common routes of giving injection viz intramuscular, intravenous, and intradermal. While giving any injections, be it for therapeutic or preventive purpose, an emergency tray should always be at hand and interns should be aware of the life-saving drugs in that tray such as adrenaline, antihistamine, and hydrocortisone. This aspect is often neglected and it is very important for management of emergencies following administration of injections. Although emergency kits are available at many centres, they are seldom used [11]. Hence health professionals should have adequate knowledge regarding the emergency drugs and their usage so that any anaphylactic situations, if arose, can be managed effectively. Most of them (93.1%) had good knowledge about this aspect. This also shows that our M.B.B.S curriculum is quite robust in educating them with adequate knowledge about various issues of injection safety. However, the study reveals that some practical aspects of injection safety were not covered during their clinical postings making them vulnerable to risk carried by unsafe injection practices.

### 4.1. IEC Intervention Package

The present study showed that the IEC intervention package was effective in significantly improving the knowledge and practical aspects of injection safety and related biomedical waste management. A highly statistically significant improvement was observed in many variables related to knowledge about injection safety as evident in Table 1. This shows that the intervention package administered to the interns after the baseline collection of information (preintervention) in the form of workshop, lectures, charts, and hands on training was helpful in improving the knowledge and practices of interns regarding various aspects of injection safety. This implies that interns who are inexperienced at the beginning of their medical career need regular reenforcement of basic knowledge and practices of universal precautions including injection safety. This should specially be emphasized in the clinical departments where chances of NSI are higher. They also need regular hands on training when they are posted in immunization clinics, wards, labour room, laboratories, accident and emergency department, and wherever they are exposed to blood and blood products.

### 4.2. Hepatitis B Immunization

After intervention, there was improvement in immunization status against hepatitis B. But there were some interns who, even after intervention, remained partially immunized or unimmunized. This raises a question, whether we should make hepatitis B vaccination mandatory for those who are partially immunized or unimmunized at the beginning of internship, when risk of exposure to need stick injury is higher. Similar result of hepatitis B immunization (66.5% and 87.5%) was reported from Vellore [7] and Tehran [8], respectively.

### 4.3. Needle Stick Injury

It was surprising to note that 6 (5.9%) interns had suffered an NSI earlier as medical students while pursuing their M.B.B.S course. This means that they were exposed to blood and blood products during their clinical, laboratory, and labour room postings during M.B.B.S. However, this problem was quite low as compared to results of other studies done on medical students in Malaysia (14.1% and 38.3%) [9, 10]. These observations suggest that safe injection practices and sound knowledge about biomedical waste management should get due attention during M.B.B.S. course and the knowledge should be imparted well before the start of clinical postings.

All the interns who had a NSI exposure received counselling and postexposure prophylaxis and also were encouraged to report all NSI.

Internship is a period where practical knowledge is imparted and opportunity is given to acquire clinical skills. At the same time, there is need to safeguard interns against avoidable occupational hazards including risk of acquiring blood borne infections like HIV, hepatitis B, and hepatitis C. Universal precautions, injection safety, and biowaste management need to be not only covered during graduate courses (MBBS) but also reinforced during internship. Regular mentoring and supervision by senior professionals are needed to inculcate safe practices.

## 5. Limitations

The limitation of our study was that we did not interview all the interns together at the end of their one-year internship, that is, on December 31, 2012. So the information regarding the actual status of hepatitis B immunization and NSI history may be underreported. This is because the postintervention assessment was carried out at the end of their two months posting in community medicine department. Hence it was carried out at different times for different batches of interns which may have introduced bias. We conducted a single workshop for the entire batch of interns at the beginning of the study. We should have conducted a workshop for every batch of interns at the beginning of their posting in community medicine for the results to be more reliable. Twelve-month recall will have questionable reliability. However, the rest of IEC package (power point presentations, videos, and charts) was given to each batch of interns.

Recall bias, particularly exposure and history of NSI during MBBS course, is another limitation of this study.

## 6. Conclusion

The baseline knowledge of interns was good in certain aspects of injection safety, namely, diseases transmitted by unsafe injections and their prevention, common routes of giving injections, and life-saving drugs in emergency tray. Their knowledge was satisfactory regarding PEP while it was poor in some other aspects like WHO criteria for safe injections, opening of glass ampoule, do not's after needle stick injury, and biomedical waste management. We conclude that there was a change in the level of knowledge as the IEC intervention package was effective in significantly improving the interns' knowledge regarding safe injection practices, prevention of NSIs, and injection related waste management. The overall status of hepatitis B immunization also improved after the postintervention assessment. Periodic reenforcement of the interns with hands on training and IEC intervention will significantly protect them from NSIs and prevent the spread of blood borne pathogens.

## Supplementary Material

The questionnaire used for data collection in this study has been enclosed. In tables 1 and 2 in the manuscript, we have applied McNemars' test we have given few examples using individual variables by making a 2X2 contigency table. In this way McNemars test was applied to all the variables in the table and the results have been summarised in tables 1 and 2.

## Figures and Tables

**Table 1:** Pretest and posttest assessment regarding injection safety after the IEC intervention.

Aspects covered in the question Knowledge about	Correct answers given (*N* = 101)		*P* value^*^
	Pretest *n* (%)	Posttest *n* (%)	
(1) Three criteria of a safe injection	23 (22.8)	75 (74.3)	<0.001
(2) Vaccine used for prevention of disease transmitted by unsafe injections, namely, Hepatitis B	93 (92.0)	96 (95.0)	0.58
(3) Three commonly used routes for giving injection	101 (100)	101 (100)	—
(4) Three common complications after giving injection	69 (68.3)	83 (82.2)	0.03
(5) Auto disable syringe	72 (71.3)	99 (98)	<0.001
(6) Cleaning of the site of injection	81 (80.2)	97 (96)	<0.001
(7) Vaccine before which spirit swab was not used	60 (59.4)	101 (100)	<0.001
(8) Correct procedure for opening a glass ampoule	10 (9.9)	43 (42.6)	<0.001
(9) Precautions to prevent needle stick injuries	49 (48.5)	93 (92.1)	<0.001
(10) Do's after a NSI	68 (67.3)	90 (89.1)	<0.001
(11) Don'ts after a NSI	24 (23.8)	75 (74.3)	<0.001
(12) Reporting a NSI	13 (12.9)	88 (87.1)	<0.001
(13) Postexposure Prophylaxis	75 (74.3)	83 (82.2)	0.21
(14) Ideal time for initiating PEP	12 (11.9)	57 (56.4)	<0.001
(15) Time after which PEP was not effective	77 (76.2)	90 (89.1)	0.01
(16) Handling needle after giving injection	27 (26.7)	57 (56.4)	<0.001
(17) Handling syringe after giving injection	10 (9.9)	39 (38.6)	<0.001

^*^ *P* value as computed by McNemar's test.

**Table 2:** Pretest and posttest assessment regarding disposal of injection related waste.

Aspects covered in the question	Correct answers given		*P* value^*^
Disposal of	Pretest *n* (%)	Posttest *n* (%)	
(1) Syringe wrapper	91 (90.1)	94 (93.1)	0.50
(2) Needle cap	54 (53.5)	67 (66.3)	0.04
(3) Empty vial	17 (16.8)	19 (18.8)	<0.001
(4) Used needles	83 (82.2)	97 (96.0)	<0.001
(5) Used syringes	57 (56.4)	80 (79.2)	<0.001
(6) Swabs	89 (88.1)	93 (92.1)	0.42
(7) Ampoule	75 (74.3)	82 (81.2)	0.28
(8) Gloves	27 (26.7)	46 (45.5)	<0.001

^*^ *P* value as computed by McNemar's test.

**Table 3:** Status of hepatitis B immunisation.

Immunisation status	Hepatitis B immunisation (*N* = 101)	
	Pretest *n* (%)	Posttest *n* (%)
Complete	67 (66.3)	78 (78.2)
Partial	22 (21.8)	20 (19.8)
Unimmunised	12 (11.9)	03 (2.0)

*P* value < 0.001.
