# Supplementary material for: Assessment of Knowledge and Practices regarding Injection Safety and Related Biomedical Waste Management amongst Interns in a Tertiary Care Teaching Hospital, Delhi
Source: Int Sch Res Notices. 2014 Oct 28;2014:670861. doi: 10.1155/2014/670861 (PMC4897080; doi:10.1155/2014/670861)
Supplement: Supplementary file 1 — The questionnaire used for data collection in this study has been enclosed. In tables 1 and 2 in the manuscript, we have applied McNemars' test we have given few examples using individual variables by making a 2X2 contigency table. In this way McNemars test was applied to all the variables in the table and the results have been summarised in tables 1 and 2. [file 670861.f1.pdf]

QUESTIONNAIRE

Proforma no: _______ Date: _____________

Name: ______________________

1. What do you understand by a safe injection? Mention 3 criteria.

2. Which are the diseases which can be contracted through unsafe injection practices?

3. Do you know of any vaccines that can be used for prevention of these diseases?

4. Name three commonly used routes for giving injection.

5. Name three common sites used for giving I/M injections.

6. Mention 3 most common complications after giving injections.

7. What do you understand by an auto-disable syringe?

8. Tick all the life saving drugs to be used in an emergency tray:

   a. Adrenaline b. Anti-biotic c. Anti-histamine d .Atropine e. Terbutaline f.Morphine g. Atenolol h. Hydrocortisone i. 25% dextrose j. Ringer Lactate solution

9. How the site should be cleaned before giving injection?

10. Do you know of any vaccine in which before its administration spirit swab should not be used?

11. How a glass ampoule for giving injections should be opened?

12. List four precautions to be used to prevent needle –stick injuries?

13. In the past one year, have you ever had any needle stick injury? Yes/ no

14. If yes, how many times?________________

15. What should be done immediately after a needle-stick injury?

16. What should not be done immediately after a needle-stick injury?

17. To whom should a needle stick injury reported in our hospital?

18. What is post-exposure prophylaxis(PEP)?

19. Ideally within how many hours PEP should be initiated? __________hrs

20. After, how many hours of accidental exposure, PEP is not effective? _________hrs

21. Are you immunized against Hepatitis B? Yes / No

22. If yes, how many doses have you received and when? ______________

23. How should you handle a needle and syringe after giving an injection?
    Needle: syringe:

24. In which of the colour coded bags the following injection related wastes should be disposed: (Black =1, Puncture proof container =2, Blue =3, Red =4, Yellow =5)

    a. Syringe wrapper:
    b. needle cap:
    c. empty vials:
    d. used needles:
    e. used syringes:
    f. swabs:
    g. Broken ampoules:

ANNEXURE 1

In Table 1 and 2, we have applied Mc Nemars Chi SquareTest which is appropriate for before after comparison in dichotomous data. We have applied the test for each variable individually and then clubbed the information in a single table to make it compact.

For example:

- Three criteria for safe injection- the variable is dichotomous with two responses- satisfactory and not satisfactory. The knowledge was considered satisfactory if the intern could mention at least two criteria for safe injection .2X2 table was prepared and McNemars test was applied.

Criteria for safe injection- Before	Criteria for safe injection- After	
	Satisfactory	Not satisfactory
satisfactory	19	4
not satisfactory	56	22

McNemar Chi Square – 43.350
Asymp. Sig. – 0.000

- Three common complications of after giving injections- this variable is also dichotomous with two responses- satisfactory and not satisfactory. The knowledge was considered satisfactory if the intern could mention at least two common complications. McNemars test was applied on the 2X2 table.

Complications- before	Complications- after	
	satisfactory	not satisfactory
satisfactory	58	11
not satisfactory	25	7

McNemar Chi Square – 4.694
Asymp. Sig. – 0.03

- In the same way, the test was applied for all the variables mentioned in Table 1 and the results were presented in a single table.

Similarly, in Table 2, correct methods of disposal of injection related waste was assessed prior to and after the intervention.

For example:

- Syringe Wrapper Disposal – dichotomous variable with two values – correct and incorrect.

Disposal of syringe wrapper - before	Disposal of syringe wrapper- after	
	correct	incorrect
correct	88	3
incorrect	6	4

**Test Statistics[a]**	
	disposal & disposal
N	101
Exact Sig. (2-tailed)	.508[b]
a. McNemar Test	
b. Binomial distribution used.	

In the same way, the values for rest of variables related to waste disposal were also calculated and they were clubbed together in table 2.
